# Effects of Temperature on Plasma Protein Binding Ratios (PPBRs) of Enrofloxacin and Ciprofloxacin in Yellow Catfish (*Pelteobagrus fulvidraco*), Grass Carp (*Ctenopharyngodon idella*), and Largemouth Bass *(Micropterus salmoides*)

**DOI:** 10.3390/ani13111749

**Published:** 2023-05-25

**Authors:** Ning Xu, Weiyu Sun, Huan Zhang, Zhi Li, Xiangzhong Luo, Xiaohui Ai, Yongzhen Ding, Bo Cheng

**Affiliations:** 1 Yangtze River Fisheries Research Institute, Chinese Academy of Fishery Sciences, Wuhan 430223, China; xuning@yfi.ac.cn (N.X.);; 2 College of Food Science and Engineering, Wuhan Polytechnic University, Wuhan 430023, China; 3 Agro-Environmental Protection Institute, Ministry of Agriculture and Rural Affairs, Tianjin 300191, China; 4 Aquatic Products Quality and Standard Research Center, Chinese Academy of Fishery Sciences, Beijing 100141, China

**Keywords:** PPBRs, enrofloxacin, ciprofloxacin, finfish, temperatures, concentrations

## Abstract

**Simple Summary:**

Enrofloxacin (EF) is a commonly used broad-spectrum antibiotic in aquaculture against Gram-negative and Gram-positive bacteria. Ciprofloxacin (CF) has similar antibacterial activities to EF and has been developed as an aquatic medicine. It is well known that only the free concentration of a drug is effective for killing challenging pathogens. Hence, the plasma protein binding ratio (PPBR) of drugs is an important parameter in clinical medicine. Although PPBRs of EF and CF have been reported in several livestock animals, little information is available on aquatic animals. Moreover, environmental temperatures may influence the PPBR of drugs used for aquatic animals. Therefore, this study investigates PPBRs of EF and CF in the plasma of several finfish species such as yellow catfish (*Pelteobagrus fulvidraco*), grass carp (*Ctenopharyngodon idella*), and largemouth bass *(Micropterus salmoides*) at different concentrations with different temperatures. The present study facilitates the design of therapeutic regimens of EF for different aquatic animals at different concentrations.

**Abstract:**

The objective of this study was to investigate the PPBRs of EF and CF in the plasma of yellow catfish, grass carp, and largemouth bass at different temperatures with different concentrations. A fast and simple ultrafiltration method was used to determine the PPBRs of EF and CF. Results showed that PPBRs of EF decreased from 37.71% to 9.66%, from 46.10% to 13.52%, and from 43.90% to 4.36% in the plasma of yellow catfish with the increase of concentration from 15 to 25 °C. The same trends of PPBRs of EF were presented in the plasma of grass carp and largemouth bass. In comparison to the data at the same concentration of EF at disparate temperatures, the PPBRs of EF at a concentration of 1 µg/mL increased from 37.71% to 46.10% and then decreased to 43.90% in the plasma of yellow catfish with elevated temperature from 15 to 25 °C. There is no obvious regularity with the rise of temperature, and the same phenomenon also were found in other concentrations and species. Meanwhile, the PPBRs of CF also decreased in the three species with the rise in concentration. Under the consistent concentration, the temperature-dependent regularities were not found in the PPBRs of CF. Overall, the increased concentration reduced the PPBRs of EF and CF in the plasma of three fish species, and the alteration in temperature only has a certain effect on the PPBRs of EF and CF.

## 1. Introduction

The plasma protein binding ratio (PPBR) is an important parameter in clinical pharmacology and the development of new pharmaceuticals in both human and veterinary medicines [1,2,3]. When drugs complete their transportation and disposition in a host organism, they can be non-specifically and reversibly bound to plasma and tissue proteins [4]. It is well-accepted that only the free drug, without binding with plasma protein, can oppose pathogens, and it decides the apparent volume of distribution to influence the drug clearance in the liver and in kidney tissues [5,6,7,8]. Therefore, a better understanding of the functions PPBRs in plasma is important for designing rational dosage regimens for drugs in order to promote therapeutic efficiency and avoid treatment failure.

Enrofloxacin (EF), a member of fluoroquinolones, is an important antibiotic for controlling some Gram-positive bacteria, most Gram-negative bacteria, mycoplasmas, and rickettsial infections [9,10,11]. It exerts its pharmacological effects by inhibiting DNA gyrase and topoisomerase IV enzymes to prevent the DNA synthesis of pathogens. This drug was approved to be used in aquaculture in several countries worldwide, such as China, Japan, South Korea, etc., mainly against *Aeromonas hydrophila*, *Flavobacterium psychrophilum*, *A. viridis*, *Vibrio parahemolyticus*, *A. salmonicida*, *Yersinia ruckeri*, etc. [12,13,14,15]. Ciprofloxacin (CF) is the main metabolite of EF and has strong antimicrobial activities against a variety of microorganisms. The study of PPBR for EF or CF possesses high significance for enhancing its therapeutic effects. Until now, the PPBR of EF has been reported in cows [16], pigs [17], broilers [18], and koalas [19]. However, no studies have been conducted on aquatic animals. It is well-known that aquatic animals are not homeothermic, and their body temperature is altered with the environmental temperature. Therefore, the shift of temperature can influence PPBR to further affect the absorption, disposition, metabolism, and excretion of drugs.

Currently, there are three main methods to detect PPBRs, such as ultracentrifugation [20], equilibrium dialysis [21], and ultrafiltration (UF) [22]. The ultracentrifugation method needs advanced instrumentation which will lead to an increase in the cost of the experiment. Equilibrium dialysis needs a long time to reach plasma equilibrium and complicates the preparation of samples and detection. Contrarily, the UF method was performed easily and fast. So, it was extensively used to determine the PPBR of drugs [23,24].

This study selected three finfish species of grass carp, yellow catfish, and largemouth bass to perform this experiment. These fish species are also commercially important cultured species in China. In 2019, their production reached 5,504,301; 509,610; and 432,058 tons [25]. Under an intensive cultured environment, these fish are easily infected by opportunistic pathogens, resulting in mass kills and huge economic losses. For these diseases, antibiotics are still the most effective method to protect them against these diseases. Through comprehensive research of references, limited information on PPBRs of EF and CF was available in grass carp, yellow catfish, and largemouth bass. Therefore, this study aimed to investigate the PPBRs of EF and CF in the plasma of these fish species at different temperatures. This study will provide fundamental data on EF and CF to help design therapeutic regimens at different temperatures.

## 2. Materials and Methods

### 2.1. Chemicals and Reagents

Centrifugal filter units with cellulose membrane of MWCO 10K (Amicon^®^Ultra-0.5 mL) were purchased from Merck Millipore Ltd. (Cork, Ireland). Determination standards of enrofloxacin (EF) with a purity of >99.2% and ciprofloxacin (CF) with a purity of >95.0% were purchased from Dr. Ehrenstorfer GmbH (Augsburg, Germany) and used for quantitative analysis. The reagents of methanol, hydrochloric acid, water, acetonitrile, n-hexane, and formic acid were bought from J.T. Baker (Philipsburg, PA, USA) and Thermo Fisher (Waltham, MA, USA). Anhydrous magnesium sulfate and sodium chloride were obtained from the Shanghai Guoyao Company (Shanghai, China). Shanghai CNW Technologies (Shanghai, China) provided 0.22-µm politetrafluoroetileno membranes, 10 mL and 15 mL centrifugal tubes, and 1.5 mL vials.

### 2.2. Animals

Thirty grass carp (average initial weight of 700.23 ± 53.48 g), fifty yellow catfish (average initial weight of 200.57 ± 21.08 g), and thirty largemouth bass (average initial weight of 400.27 ± 30.55 g) were obtained from the breeding base of Yangtze River Fisheries Research Institute (Wuhan, China). Fish were reared in tanks (400 L per tank) receiving water (26 L/min). Water parameters were detected daily and kept to the following extent: total ammonia nitrogen (T-AN) concentrations at ≤0.71 mg/L, dissolved oxygen (DO) concentrations at 6.3–6.8 mg/L, nitrite concentrations at <0.067 mg/L, and pH at 6.9 ± 0.3. The water temperature was 25 ± 0.5 °C. Blood was drawn from each fish with a 5 mL heparinized syringe and centrifuged at 3000× *g* for 10 min. The plasma of all animals was decanted into a 10 mL centrifuge tube and stored at −20 °C. All experimental protocols were approved by the Fish Ethics Committee of Yangtze River Fisheries Research Institute, Chinese Academy of Fishery Sciences, Wuhan, China.

### 2.3. Ultrafiltration Methodology

The detailed procedures and the pretreatment of the ultrafiltration unit (UFU) were in line with the previous reports [26,27]. In brief, the nonspecific absorption (NSB) of UFU was first checked before and after pretreatment of 5% Tween 80. A half milliliter of EF or CF solution with a concentration of 1, 5, and 10 µg/mL was added to the UFU untreated by 5% Tween 80. All samples of EF or CF were pre-incubated for 1 h at 25 °C inside the centrifuge (HITACHI, CT15RE). Firstly, 100 μL of the sample was used to evaluate the initial concentration before centrifugation. Afterward, All drug solutions were centrifugated at 4700× *g* for 5 min to get filtrate for assay using high-performance liquid chromatography (HPLC). To reduce the NSB of UFU, 100 μL of 5% Tween 80 was used to treat UFU at 25 °C for 5 min, and 0.5 mL of physiological saline was used to wash off Tween 80. The NSB of UFU was repeatedly determined again in light of the above-mentioned method.

**Figure 1:**
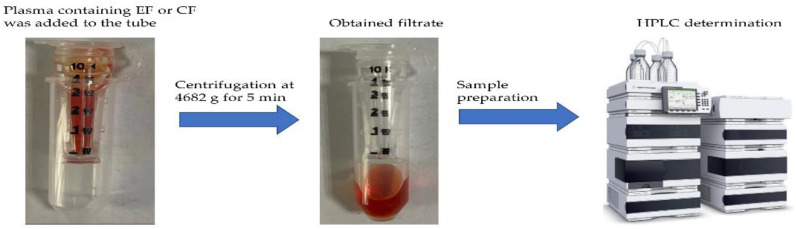
The procedure for PPBR determination.

After the evaluation of the NSB, the PPBR of EF or CF was measured in the plasma of yellow catfish, grass carp, and largemouth bass. Plasma with different concentrations of EF or CF was added to the UFU treated by Tween 80, and they were incubated in the centrifuge at 15, 20, and 25 °C for 1 h. Before centrifugation, 100 μL of the plasma containing drug was pipetted from the UFU to evaluate the level of EF or CF. All samples were centrifugated at 4700× *g* for 5 min to get filtrate. The real procedures were displayed in Figure 1.

### 2.4. Sample Preparation

The samples of sterile physiological saline were detected without extraction by HPLC. As for the samples of plasma, 0.1 mL of plasma was decanted into a 10 mL centrifugation tube. The extraction method is the same as the reported literature [28].

### 2.5. HPLC Analysis and Validation

Samples of plasma and sterile physiological saline were determined by an Agilent HPLC 1260 Infinity II system (Santa Clara, CA, USA), consisting of a fluorescent detector, a Quaternary solvent manager with a Quaternary solvent pump, and a sampler manager with an autosampler. Isocratic elution was used to separate EF and CF by a column of Poroshell 120 EC-C18 (2.7 μm, 4.6 mm × 100 mm) with a temperature of 35 °C. The proportion of the mobile phase was 82:18 (water containing 0.2% formic acid and pure acetonitrile, *v*/*v*), following the flow rate of 0.8 mL/min. The excitation wavelength was 280 nm. The emission wavelength was 450 nm.

The HPLC method was validated by the linearity, recovery, precision, specificity, limit of detection (LOD), and limit of quantification (LOQ). The detailed procedures are according to the published references [28,29,30].

### 2.6. Calculation of NSB and PPBR

Values of *NSB* were calculated by the equation below:

(1) $$NSB=\left(\frac{C_{1}-C_{2}}{C_{1}}\right)\times 100\%$$

where *C_1_* is the EF or CF’s level in the original physiological saline, and *C_2_* is the EF or CF’s level in the filtrate.

The *PPBRs* of EF and CF were computed following the equation below:

(2) $$PPBR=\left(\frac{C_{P}-C_{UF}}{C_{P}}\right)\times 100\%$$

where *C_P_* is the EF or CF’s level in the original plasma, and *C_UF_* is the EF or CF’s level in the filtrate.

### 2.7. Statistical Analysis

The concentrations of EF and CF were expressed as mean ± SD. Statistical differences of the PPBRs of EF or CF in the plasma at different concentrations and temperatures were analyzed using a one-way ANOVA analysis using SPSS Statistics 23.0 software.

## 3. Results

### 3.1. HPLC Analysis

The values of the LOD for EF and CF were 0.005 µg/mL in physiological saline and the plasma of the three species. The values of the LOQ for EF and CF were 0.01 µg/mL in physiological saline and plasma. The matrix-fortified calibration curves presented good linearity with a coefficient of correlation of R-value ≥ 0.999 (Table 1). The mean recovery rates of EF were 84.70–109.66%, 92.20–118.53%, and 90.09–93.09% in the plasma of yellow catfish, grass carp, and largemouth bass, respectively (Table 2). Their percentages of relative inter-day standard deviations were 0.47–2.28%, and intra-day precisions were 1.03–9.78%. The mean recovery rates of CF were 81.16–101.77%, 92.62–106.23%, and 87.75–94.32% in the plasma of yellow catfish, grass carp, and largemouth bass, respectively (Table 2). Their percentages of relative inter-day standard deviations for were 1.21–3.29%, and intra-day precisions were 2.30–9.53%.

### 3.2. Non-Specific Absorption

Table 3 showed the NSB ratios of the UFU for EF and CF before and after treatment with the Tween 80 solution (5%). The NSB ratios of the UFU for EF and CF were 82.05–99.24% and 72.13–98.21% before pretreatment with 5% Tween 80 at concentrations of 1, 5, and 10 µg/mL. After pretreating UFU with the Tween 80 solution (5%), the NSB ratios of the UFU for EF and CF were reduced to 0.52–16.14% and 4.08–10.47% at the same concentrations.

### 3.3. PPBRs of EF and CF at Different Concentrations at the Same Temperature

Table 4 showed the PPBRs of EF and CF at different concentrations in the plasma of yellow catfish, grass carp, and largemouth bass. Superscript letters were marked to represent statistical significance. Results showed that the PPBRs of EF decreased from 37.71% to 9.66% in yellow catfish plasma, from 60.52% to 16.95% in grass carp plasma, and from 35.11% to 9.30% in largemouth bass with increased concentration from 1 to 10 µg/mL at 15 °C. At 20 and 25 °C, the PPBRs of EF presented the same trends at the same temperature in the plasma of yellow catfish, grass carp, and largemouth bass. Statistical analysis showed that the differences in the PPBRs were significant among different concentrations of plasma of all three species. As for the PPBRs of CF, its values declined from 27.02% to 11.46% in yellow catfish, from 28.30% to 10.86% in grass carp, and from 21.64% to 15.85% in largemouth bass, with the rise of CF concentration from 1 to 10 µg/mL at 15 °C. At 20 and 25 °C, the PPBRs of CF also presented an identical reduced trend in the three species. The PPBRs of CF were statistically significant among different concentrations of CF except for yellow catfish at 25 °C and grass carp at 20 °C.

### 3.4. PPBRs of EF and CF at Different Temperatures at the Same Concentration

Table 5 showed the PPBRs of EF and CF at different temperatures at the same concentrations in the plasma of yellow catfish, grass carp, and largemouth bass. Superscript letters were marked to represent statistical significance. In yellow catfish plasma, the PPBRs of EF at a concentration of 1 µg/mL were increased from 37.71% to 46.10% and then decreased to 43.9% with elevated temperature from 15 to 25 °C. At a concentration of 5 µg/mL, the PPBRs of EF decreased from 22.53% to 17.29%. At a concentration of 10 µg/mL, the PPBRs of EF were first increased from 9.66% to 13.52% and then decreased to 4.36%. One-way ANOVA analysis showed statistical significance among the PPBRs at different temperatures except for the concentration of 5 µg/mL. In the grass carps and largemouth basses, we also did not find obvious regularities of EF’s PPBRs along with the rise of temperature. As for CF, the PPBRs at 1 µg/mL of CF were first decreased from 27.02% to 24.22% and then increased to 27.85% with the ascent of temperature from 15 to 25 °C. At a concentration of 5 µg/mL, the PPBRs were first reduced from 14.39% to 12.14% and then increased to 23.36%. At a concentration of 10 µg/mL, the PPBRs also first declined from 11.46% to 7.04% and then increased to 21.64%. In the plasma of grass carp and largemouth bass, the PPBRs of CF did not present temperature-dependent properties as well.

## 4. Discussion

In the present study, we adopted the UF method to examine the PPBRs of EF and CF at different concentrations at different temperatures in the plasma of yellow catfish, grass carp, and largemouth bass. UF is a commonly used method to determine the PPBRs of drugs in human and veterinary medicine. Compared to other methods of equilibrium dialysis and ultracentrifugation, UF is a fast, simple, and accurate method to save time, workforce, and research funding; however, the NSB exists for some drugs, which influences the determination of PPBRs. The main factors are the lipophilicity and molecular mass of drugs [22]. If the lipophilicity of a drug is higher, the NSB will be more likely to occur. It can be overcome by presaturation of the filter with an unlabeled compound, but the free concentration of the drug will be overestimated due to the uncontrolled desorption and displacement of adsorbed compounds. Regarding molecular mass, when it is more than 500 D, it might generate a molecular sieving effect, leading to lower medicine concentration in the filter [22]. It is reported that the UFU was pretreated with Tween 80 or benzalkonium chloride, causing a reduction in the NSB. Tween 80 could impede potential hydrophobic interaction for neutral and acidic compounds, for example, hydrocortisone, etoposide, and ibuprofen [26]. Benzalkonium chloride could block potential ionic interaction for basic compounds, such as vinblastine and propranolol [26]. EF and CF belong to acidic compounds category. The use of Tween 80 to reduce the NSB brought good effects for EF and CF in the plasma of yellow catfish, grass carp, and largemouth bass. Moreover, UF also has other disadvantages, including the leakage of protein, the sieve effect, the Gibbs—Donnan effect, the need for rigorous control of pH and temperature, and the effect of the volume ratio of the ultrafiltrate [31,32]. These contents are beneficial to explain particular phenomena in the studies of PPBRs.

The result demonstrated that the PPBRs of EF and CF decreased with the rise of EF and CF concentration in the plasma of the three fish species. This is consistent with the previous study. The protein binding of most drugs is a saturable process [26]. To a certain extent, the protein binding ratio is increased along with the rise of fortified concentration of the drug but saturated after getting a concentration threshold. However, there is also an abnormal phenomenon for protein binding. The PPBRs of tetracyclines increased all the time with the ascent of concentration caused by the atypical nonlinearity of the PPBR for tetracyclines. In previous studies, this phenomenon was also found in the PPBR of doxycycline, minocycline, and tigecycline [33,34,35]. The reason may be attributable to tetracyclines strongly chelating divalent cations to form complexes that are irreversible. Through extensive research of the literature, we found that the most important transport proteins with a molecular weight of 66 KD (serum albumin (SA)) possess a high capacity for binding with endogenous and exogenous compounds in plasma. SA comprised three homologous a-helical domains (I–III), and Sudlow’s sites in subdomains IIA and IIIA are the main sites for binding drugs, but the third site is bonded by digoxin [36]. SA could combine with acidic or basic drugs and many endogenous substances in the plasma by hydrogen bonding, ionic interactions, van der Waals dispersion and hydrophobic forces, and other attractive forces as well. Particularly, binding site IIIA is more sensitive to the basic drug up to saturation of site IIA, but it can bind acidic compounds [36]. Drugs or other endogenous compounds can compete for the same binding site in albumin; hence, co-administered drugs or endogenous compounds may strengthen the free fraction of the target drug by performing a competition for the binding to the same protein site, then a displacement of the drug with the limited binding affinity occurs and is non-bound. This hypothesis has been proved by the study of warfarin being co-administered with acidic antibiotics or in the case of existing high concentrations of endogenous compounds [37]. Therefore, it is an effective method to help enhance the free concentration of drugs possessing high PPBRs in aquatic animals.

Although the PPBRs were scarcely determined in fish, they are reported in many land animals. In cows, the PPBRs of EF and CF were estimated to be 59.40% and 49.60% using the ultrafiltration (UF) method at 10 °C after administering EF at a dose of 5 mg/kg by intravenous injection. Meanwhile, the PPBRs of EF and CF were estimated to be 60.80% and 33.80% in beef steers [16]. These studies indicated that the PPBR of CF was lower than that of EF at the same concentration, which is consistent with the result in our study. In pigs, EF’s PPBR was evaluated to be 31.10% and 37.13% for high and low concentrations using the UF method [17], suggesting that the PPBR at a high concentration is lower than that at a low concentration. This may be due to the PPBR of the drug being affected by process of saturation. This phenomenon was also found in the present study. Otherwise, the PPBR was determined to be 10.6% in freshwater crocodiles [38]. This is similar to the result at high concentrations in our study. In broilers, the PPBR of EF was assessed to be 22.70% [18]. In koalas, the PPBRs of EF were evaluated to be 55.40% using the ultracentrifugation method after incubating the plasma of koalas with EF (1 µg/mL) for 30 min [19]. In different species, the PPBRs of EF and CF are different partly due to the distinct abilities of protein in different species of animals.

Environmental temperature can alter the temperature of fish bodies due to their being poikilothermal animals. Hence, in this study, we consider determining the PPBRs of EF and CF in the plasma at different temperatures. We found that the PPBRs of EF decreased with the temperature rise. Therefore, the temperature has an effect on the PPBRs of EF in the plasma of aquatic animals. The PPBRs of EF at 15 °C were higher than that at 25 °C in the plasma of grass carp at 1, 5, and 10 µg/mL. The PPBRs of EF at 15 °C were higher than that at 25 °C in the plasma of yellow catfish at 5 and 10 µg/mL except for 1 µg/mL. This finding is consistent with the report of doxycycline in grass carp and yellow catfish [35]. These suggested that, under higher temperatures, a smaller amount of EF was bound to plasma protein. In other words, the antimicrobial effect of EF may be better at warm temperatures that the low temperature in grass carp and yellow catfish. However, this regularity was not found in the PPBRs of EF in largemouth bass and the PPBRs of CF in the three species. The exact reason was not found. The species difference may be a partial reason.

## 5. Conclusions

In summary, we observed that as the concentration increased, the PPBRs of EF and CF decreased at the same temperature in the plasma of the studied finfish species. This trend was not presented in the PPBRs of EF in the plasma of largemouth bass and the PPBRs of CF. Therefore, the temperature only has a certain effect on the PPBRs of EF. Hence, these conclusions can directly guide the clinical usage of EF during the course of treatment in yellow catfish, grass carp, and largemouth bass.

## Figures and Tables

**Table 1:** Standard curve equations and correlation coefficients of EF and CF in physiological saline and the plasma of yellow catfish, grass carp, and largemouth bass (*n* = 3).

Drugs	Matrixes	Concentration Ranges (μg/mL)	Regressed Equations	Correlation Coefficients^®^
Enrofloxacin (EF)	Physiological saline	0.01~1	y = 0.7936x + 0.2670	0.9999
	Yellow catfish		y = 0.7261x + 5.7537	0.9992
	Grass carp		y = 0.9575x + 2.6615	0.9993
	Largemouth bass		y = 0.960–x − 0.9355	0.9998
Ciprofloxacin (CF)	Physiological saline		y = 0.1387x + 0.4517	0.9999
	Yellow catfish		y = 0.1301x + 1.5801	0.9987
	Grass carp		y = 0.181–x − 00.2204	0.9992
	Largemouth bass		y = 0.175–x − 00.9423	0.9996

**Table 2:** Accuracy and precision of the analytical method for EF and CF in the plasma of yellow catfish, grass carp, and largemouth bass (*n* = 3).

Drugs	Plasma	Spiked Concentrations (μg/mL)	Recoveries (%)	Intra-Day RSDs (%)	Inter-Day RSDs (%)
Enrofloxacin (EF)	Yellow catfish	0.02	109.66 ± 5.65	2.10	9.32
		0.1	90.17 ± 7.45	1.56	6.78
		1	84.70 ± 1.79	2.28	2.59
	Grass carp	0.02	118.53 ± 2.30	1.02	9.78
		0.1	92.20 ± 2.33	0.47	2.90
		1	93.18 ± 0.60	0.53	1.03
	Largemouth bass	0.02	90.09 ± 2.74	1.91	3.93
		0.1	91.36 ± 1.45	2.12	4.89
		1	93.09 ± 0.40	2.11	2.80
Ciprofloxacin (CF)	Yellow catfish	0.02	101.77 ± 7.24	2.23	5.67
		0.1	97.82 ± 0.68	1.21	2.47
		1	81.16 ± 0.69	2.43	4.60
	Grass carp	0.02	106.23 ± 0.58	3.29	4.06
		0.1	92.62 ± 1.43	2.21	2.30
		1	94.72 ± 1.44	1.44	2.91
	Largemouth bass	0.02	87.75 ± 4.48	2.23	3.89
		0.1	94.20 ± 0.58	1.35	9.53
		1	94.32 ± 1.27	1.90	2.83

Note: RSD, relative standard deviation.

**Table 3:** Nonspecific absorption percentages of EF and CF for ultrafiltration membrane by no pretreatment and pretreatment with 5% Tween 80 (*n* = 3).

Drug	Spiked Concentration (μg/mL)	No Pretreatment NSB (%)	Pretreatment NSB (%)
Enrofloxacin	1	99.24	0.52
	5	93.33	9.66
	10	82.05	16.14
Ciprofloxacin	1	98.21	4.08
	5	91.06	8.02
	10	72.13	10.47

Note: NSB, nonspecific absorption.

**Table 4:** Statistical analysis of plasma protein binding ratios of EF and CF at different concentrations under the same temperature (mean ± SD, *n* = 3).

Drugs	Fish Species	Spiked Concentrations (μg/mL)	PPBRs (%)		
			15 °C	20 °C	25 °C
Enrofloxacin (EF)	Yellow catfish	1	37.71 ± 3.71 ^a^	46.10 ± 3.13 ^a^	43.90 ± 3.62 ^a^
		5	22.53 ± 6.86 ^b^	21.24 ± 6.93 ^b^	17.29 ± 0.69 ^b^
		10	9.66 ± 4.09 ^c^	13.52 ± 4.74 ^b^	4.36 ± 0.58 ^c^
	Grass carp	1	60.52 ± 0.99 ^a^	51.27 ± 1.56 ^a^	50.09 ± 2.06 ^a^
		5	33.77 ± 0.07 ^B^	22.07 ± 1.19 ^B^	23.36 ± 3.86 ^B^
		10	16.95 ± 1.81 ^C^	8.34 ± 2.29 ^C^	5.52 ± 1.99 ^C^
	Largemouth bass	1	35.11 ± 5.18 ^a^	37.93 ± 6.11 ^a^	39.12 ± 1.72 ^a^
		5	14.39 ± 0.61 ^b^	17.46 ± 2.82 ^b^	23.20 ± 5.87 ^b^
		10	9.30 ± 5.15 ^C^	10.84 ± 1.08 ^C^	7.77 ± 2.10 ^C^
Ciprofloxacin (CF)	Yellow catfish	1	27.02 ± 2.31 ^a^	24.22 ± 0.91 ^a^	27.85 ± 2.14 ^a^
		5	14.39 ± 1.03 ^b^	12.14 ± 0.36 ^b^	23.36 ± 0.96 ^a^
		10	11.46 ± 1.35 ^b^	7.04 ± 1.36 ^b^	21.64 ± 3.34 ^a^
	Grass carp	1	28.30 ± 0.80 ^a^	27.69 ± 1.05 ^a^	28.09 ± 1.79 ^a^
		5	18.22 ± 4.13 ^b^	21.61 ± 1.00 ^a^	24.65 ± 1.95 ^a^
		10	10.86 ± 2.85 ^b^	17.29 ± 1.15 ^a^	12.48 ± 1.07 ^b^
	Largemouth bass	1	21.64 ± 1.34 ^a^	24.21 ± 3.89 ^a^	33.72 ± 2.43 ^a^
		5	17.36 ± 2.70 ^b^	20.88 ± 3.35 ^b^	14.53 ± 2.30 ^b^
		10	15.85 ± 2.43 ^b^	10.70 ± 4.09 ^b^	10.95 ± 3.91 ^b^

Note: PPBRs at different concentrations at the same temperature marked with different lowercase letters show significant differences between groups (*p* < 0.05). If the data were marked with the same lowercase letters, the difference between groups is not significant (*p* > 0.05). If the data were marked with capital letters, the difference between groups is highly significant (*p* < 0.01). SD: standard deviation.

**Table 5:** Statistical analysis of plasma protein binding ratios of EF and CF at different temperatures under the same concentration (mean ± SD, *n* = 3).

Drugs	Fish Species	Temperature (°C)	PPBRs (%)		
			1 μg/mL	5 μg/mL	10 μg/mL
Enrofloxacin	Yellow catfish	15	37.71 ± 3.71 ^b^	22.53 ± 6.86 ^a^	9.66 ± 4.09 ^ab^
		20	46.10 ± 3.13 ^a^	21.24 ± 6.93 ^a^	13.52 ± 4.74 ^a^
		25	43.90 ± 3.62 ^ab^	17.29 ± 0.69 ^a^	4.36 ± 0.58 ^b^
	Grass carp	15	60.52 ± 0.99 ^a^	33.77 ± 0.07 ^a^	16.95 ± 1.81 ^a^
		20	51.27 ± 1.56 ^b^	22.07 ± 1.19 ^b^	8.34 ± 2.29 ^b^
		25	50.09 ± 2.06 ^b^	23.36 ± 3.86 ^a^	5.52 ± 1.99 ^B^
	Largemouth bass	15	35.11 ± 5.18 ^a^	14.39 ± 0.61 ^a^	9.30 ± 5.15 ^a^
		20	37.93 ± 6.11 ^a^	17.46 ± 2.82 ^ab^	10.84 ± 1.08 ^a^
		25	39.12 ± 1.72 ^a^	23.20 ± 5.87 ^b^	7.77 ± 2.10 ^a^
Ciprofloxacin	Yellow catfish	15	27.02 ± 2.31 ^a^	14.39 ± 1.03 ^b^	11.46 ± 1.35 ^b^
		20	24.22 ± 0.91 ^a^	12.14 ± 0.36 ^b^	7.04 ± 1.36 ^b^
		25	27.85 ± 2.14 ^a^	23.36 ± 0.96 ^a^	21.64 ± 3.34 ^a^
	Grass carp	15	28.30 ± 0.80 ^a^	18.22 ± 4.13 ^a^	10.86 ± 2.85 ^b^
		20	27.69 ± 1.05 ^a^	21.61 ± 1.00 ^a^	17.29 ± 1.15 ^a^
		25	28.09 ± 1.79 ^a^	24.65 ± 1.95 ^a^	12.48 ± 1.07 ^b^
	Largemouth bass	15	21.64 ± 1.34 ^a^	17.36 ± 2.70 ^a^	15.85 ± 2.43 ^a^
		20	24.21 ± 3.89 ^a^	20.88 ± 3.35 ^a^	10.70 ± 2.09 ^a^
		25	33.72 ± 2.43 ^a^	14.53 ± 2.30 ^b^	10.95 ± 3.91 ^a^

Note: PPBRs at different concentrations at the same temperature marked with different lowercase letters show significant differences between groups (*p* < 0.05). If the data were marked with the same lowercase letters, the difference between groups is not significant (*p* > 0.05). If the data were marked with capital letters, the difference between groups is highly significant (*p* < 0.01). SD: standard deviation.

## Data Availability

Not applicable.

## Abbreviations

CF: ciprofloxacin; EF, enrofloxacin; HPLC, high-performance liquid chromatography; LOD, limit of detection; LOQ, limit of quantification; NSB, nonspecific absorption; PPBR, plasma protein binding ratio; RSD, relative standard deviation; SA, serum albumin; SD, standard deviation; UF, ultrafiltration; UFU, ultrafiltration unit.
